# Draft Genome Sequences of Histamine- and Non-Histamine-Producing *Photobacterium* Strains

**DOI:** 10.1128/genomeA.01008-16

**Published:** 2016-09-22

**Authors:** Kristin Bjornsdottir-Butler, Maria Sanchez Leon, Paul V. Dunlap, Ronald A. Benner

**Affiliations:** a FDA, Division of Seafood Science and Technology, Gulf Coast Seafood Laboratory, Dauphin Island, Alabama, USA; b FDA, Division of Public Health Informatics and Analytics, College Park, Maryland, USA; c University of Michigan, Ann Arbor, Michigan, USA

## Abstract

Histamine-producing bacteria (HPBs) have recently been identified from the marine environment. The identification and characterization of HPBs is important to developing effective mitigation strategies for scombrotoxin fish poisoning. We report here the draft genomes of seven histamine-producing and two non-histamine-producing marine *Photobacterium* strains.

## GENOME ANNOUNCEMENT

In the United States, rapid chilling of fish and fish products to ≤4.4°C is recommended to control histamine (scombrotoxin) formation (1). *Photobacterium* spp. are ubiquitous in the marine environment and have been isolated from the edible portion of scombrotoxin-containing fish, for example, tuna and mahi-mahi (2, 3). Some strains are able to grow and produce toxic concentrations of histamine at low temperatures (3–5). Indeed, several outbreaks of scombrotoxin poisoning have been linked to *Photobacterium* spp. (5). In addition, variability in histamine production between strains of the same species has been noted (3). Therefore, psychrotrophic histamine-producing *Photobacterium* spp. are of particular concern in the occurrence of scombrotoxin-associated illnesses.

The *Photobacterium* strains sequenced in this study were histamine-producing isolates of *Photobacterium aquimaris* (BS-1 [6] and BS-2 [6]), *Photobacterium phosphoreum* (AK-3 [7], FS-1.1 [7], and FS-3.1 [7]), and *Photobacterium kishitanii* (DSM-2167, *calba*.1.1 [7]) and non-histamine-producing isolates of *Photobacterium aquimaris* (DSMZ-23343), and *Photobacterium damselae* (BT-6 [8]), isolated from various marine sources. The strains were sequenced to confirm their species identifications and the presence or absence of the histidine decarboxylase gene (*hdc*, involved in formation of histamine).

The genomes were sequenced using an Ion PGM sequencer and the Ion OneTouch 2 system with 400-bp reads (Life Technologies, Frederick, MD, USA). Briefly, for DNA purification, single colonies were incubated in 5 ml of Luria 70% seawater (LSW-70) (9) at 20°C with shaking at 200 rpm for 24 h. DNA was extracted with the DNeasy blood and tissue kit according to the manufacturer’s instructions (Qiagen, Valencia, CA, USA). DNA concentrations were determined using a Qubit 2.0 fluorometer with the Qubit dsDNA HS assay kit according to the manufacturer’s instructions (Life Technologies). DNA was enzymatically fragmented using the Ion Xpress Plus fragment library kit (Life Technologies), and size was determined with E-Gel SizeSelect 2% agarose gels in an E-Gel iBase unit (Life Technologies). The template for the Ion Torrent PGM instrument was prepared with the Ion PGM Template OT2 400 kit and sequenced with the Ion PGM sequencing kit on an Ion 318 V2 chip according to the manufacturer’s instructions (Life Technologies). For each isolate, the genomic sequence single-pass reads were *de novo* assembled using SPAdes software (10) and annotated using the NCBI Prokaryotic Genome Annotation Pipeline (http://www.ncbi.nlm.nih.gov/genome/annotation_prok) (11). Through annotation, 3,971 to 4,212, 4,149 to 4,405, 4,396 to 4,637, and 3,986 genes were identified for the *P. aquimaris*, *P. phosphoreum, P. kishitanii*, and *P. damselae* isolates, respectively. The identification of the isolates and the presence of the *hdc* gene was confirmed in the histamine-producing isolates of *P. aquimaris* (BS-1 and BS-2), *P. kishitanii* (DSM-2167 and *calba*.1.1) but not in the histamine-producing isolates of *P. phosphoreum* (AK-3, FS-1.1, FS-3.1).

### Accession number(s).

The draft genome sequences of the three *P. aquimaris*, two *P. kishitanii*, three *P. phosphoreum*, and one *P. damselae* isolate are available in GenBank under the accession numbers LZEZ00000000, LZFA00000000, LZFB00000000, LZFC00000000, LZFD00000000, LZFE00000000, LZFF00000000, LZFG00000000, and LZFH00000000.
